# GPI: An indicator for immune infiltrates and prognosis of human breast cancer from a comprehensive analysis

**DOI:** 10.3389/fendo.2022.995972

**Published:** 2022-09-28

**Authors:** Jie Zeng, Jianing Yi, Siyi Tan, Yuanjun Zeng, Lianhong Zou, Chaojie Zhang, Luyao Liu, Pingyong Yi, Peizhi Fan, Jie Yu

**Affiliations:** ^1^ Department of Breast and Thyroid Surgery, Hunan Provincial People’s Hospital, The First Affiliated Hospital of Hunan Normal University, Changsha, China; ^2^ Department of Medical Laboratory, Huazhi Medical Laboratory Co., Ltd, Changsha, China; ^3^ Department of Pathology, Hunan Provincial People’s Hospital, The First Affiliated Hospital of Hunan Normal University, Changsha, China; ^4^ Institute of Translational Medicine, Hunan Provincial People’s Hospital, The First Affiliated Hospital of Hunan Normal University, Changsha, China; ^5^ Department of Oncology, Changsha Jing Kai Hospital, Changsha, China

**Keywords:** Glucose-6-phosphate isomerase (*GPI*), biomarker, prognosis, breast cancer, immune infiltrates, cell cycle

## Abstract

Glucose-6-phosphate isomerase (*GPI*) plays an important part in gluconeogenesis and glycolysis through the interconversion of d-glucose-6-phosphate and d-fructose-6-phosphate, and its clinical significance still remains unclear in breast cancer (BRCA). We analyzed the expressions of *GPI* in BRCA patients to determine prognostic values. Our results showed that the expression levels of *GPI* were upregulated in BRCA patients, and a high *GPI* expression is correlated with poor overall survival (OS) in BRCA. At the same time, a high *GPI* expression is correlated with poor clinicopathological characteristics, such as stage III, over 60 years old, N3, HER2 negative, and estrogen receptor (ER) positive. Further analysis of the influence of *GPI* on the prognosis of BRCA suggested that 50 genes and 10 proteins were positively correlated with *GPI*, and these genes and proteins were mainly involved in cell cycle signaling pathways. In addition, in this study, we observed that *GPI* was closely related to *N* ^6^-methyladenosine (m6A) RNA methylation modification and immune cell infiltration and ferroptosis-related gene expression in BRCA, and there was a difference in m6A RNA methylation alterations, immune cell infiltration, and ferroptosis-related gene expression between the high *GPI* expression group and the low *GPI* expression group. Finally, we found that *GPI* in BRCA had 2.6% gene alterations, and BRCA patients with gene alteration of *GPI* had a poor prognosis in disease-free survival (DFS). Altogether, our work strongly suggested that *GPI* may serve as a new prognostic biomarker for BRCA patients.

## Highlights

1. The expression of *GPI* affects the survival rate of breast cancer patients
2. *GPI* is closely related to cell cycle regulatory genes
3. *GPI* expression is correlated to immune cell infiltration
4. *GPI* expression is associated with m6A RNA methylation regulation and gene alteration
5. *GPI* expression is associated with ferroptosis genes in BRCA

## Introduction

Female breast cancer has now been the most commonly occurring cancer in the world in 2020, with approximately 2.3 million new cases, accounting for 11.7% of overall cancer cases. Among women, breast cancer is the leading cause of cancer death, with 685,000 deaths (1). Recently, surgery, radiotherapy, chemotherapy, immunotherapy, and targeted drug therapy have made a great amount of progress in treating breast cancer (2, 3). However, there is still a poor survival rate for some metastatic breast cancer patients, and only one-fifth of these patients survive over 5 years (4). Due to tumor heterogeneity, the current prognostic biomarkers for breast cancer have some drawbacks (5). Therefore, it is imperative to find more effective prognostic biomarkers for optimizing the treatments of breast cancer.

Carcinogenesis, progression, and metastasis are involved in epigenetic and genetic alterations, including gene mutations, variation of metabolic enzymes (3), alteration of the tumor microenvironment (5), and DNA methylation change (6). As one of the glucose-metabolizing enzymes, *GPI* plays an important part in gluconeogenesis and glycolysis through the interconversion of d-glucose-6-phosphate and d-fructose-6-phosphate (7, 8). Furthermore, *GPI* can be released to the outside of cells functioning as a cytokine or growth factor (9). In patients, the levels of *GPI* are significantly higher in both serum and synovial fluid (9). In recent years, it has been reported that there was an aberrant expression of *GPI* in several cancers (10–12). A high expression of *GPI* in lung adenocarcinoma and renal cell carcinoma was related to poor prognosis (10, 11). However, the clinical significance of *GPI* in human breast cancer remains unclear. Bioinformatics analysis has been applied to survey the role of *GPI* in cancers (10).

In the present study, according to the analysis of *GPI* gene expressions in published databases, we evaluated the relationship between *GPI* expression and the prognosis of breast cancer patients and conducted an analysis of the influence of *GPI* on the prognosis. Our results indicated that *GPI* can be used as a biomarker to predict the prognosis of breast cancer patients.

## Materials and methods

### Ethics statement

The study has been authorized by the Ethics Committee of Hunan Provincial People’s Hospital/The First Affiliated Hospital of Hunan Normal University (document no. 2022-49) and practiced in accordance with the research principles described in the Declaration of Helsinki.

### Tumor immune estimation resource database

TIMER2 (Tumor Immune Estimation Resource, version 2, http://timer.cistrome.org/) (13) was used to analyze the expression profiling of *GPI* between tumor types and adjacent normal tissues, and the relationship between *GPI* expression and immune cell infiltration was evaluated.

### RNA-sequencing data of *GPI* in human breast cancer

The RNA-Seq expression data of *GPI* in breast cancer (BRCA) come from The Cancer Genome Atlas (TCGA) (https://portal.gdc.cancer.gov/). Therefore, many tumor data and adjacent normal tissue data were retained. For some tumors without normal or with highly limited normal tissues, TCGA normal and Genotype-Tissue Expression (GTEx) data were matched. The samples selected included *GPI* gene expression data and associated clinical information, such as age, pathological stage, race, and histological type.

### Immunohistochemistry

A total of 20 samples of paraffin-embedded breast cancer tissues and their matched paracancerous tissues were gained from breast cancer patients who were treated at Hunan Provincial People’s Hospital/The First Affiliated Hospital of Hunan Normal University. Tumor tissue and its adjacent normal tissues were made into 4-mm paraffin sections, and these sections were treated with primary rabbit monoclonal antibodies of *GPI* (1:500 dilution; Cell Signaling Technology, Danvers, MA, USA) at 4°C overnight. After being washed with phosphate-buffered saline (PBS), each section was treated with horseradish peroxidase (HRP)-labeled goat anti-rabbit as a secondary antibody (1:1500 dilution; Beyotime, Shanghai, China) for 2 h at 37°C. The sections were stained with 3,3′-diaminobenzidine (DAB) and counterstained with hematoxylin (Beyotime, Shanghai, China). A semiquantitative integration method was used to analyze staining intensity using ImageJ software.

### Survival analysis

An R package was used to estimate the correlation between *GPI* expression and the survival rate of different clinical features in BRCA patients, and the hazard ratio (HR) and log-rank p-value of the 95% confidence interval were calculated.

### Functional Enrichment Analysis

To investigate the biological processes that *GPI* may be involved in, using the Gene Expression Profiling Interactive Analysis (GEPIA) database (http://gepia.cancer-pku.cn/index.html) (14), an analysis of the 25 genes with positive and negative correlations of *GPI* was carried out. The genes were enriched by Gene Ontology (GO) containing molecular function (MF), biological processes (BP), and cellular constituents (CC). Kyoto Encyclopedia of Genes and Genomes (KEGG) pathway analyses were visualized by the R package “ggplot2”. A corrected p < 0.05 was considered to be statistically significant. In addition, the “clusterProfiler” R package was applied to conduct GO enrichment analysis. The data for BP, CC, MF, and KEGG were visualized in Network.

### STRINGS analysis

STRINGS (www.string-db.org) (15) is a network tool for the analysis of protein–protein interaction (PPI). In this study, we performed a PPI analysis of *GPI* to explore their functions in human breast cancer. The basic settings were as follows: meaning of network edges: “evidence”, active interaction sources: “experiments and textmining”, the minimum required interaction score: “medium confidence (0.400)”, and max number of interactors to show: “no more than 10 interactors” in 1st shell.

### Genetic alteration analysis

By the cBioPortal tool (https://www.cbioportal.org/) (16, 17), the data of gene amplification, missense mutation, truncating mutation, and deep deletion in BRCA of all TCGA tumors were collected. The mutated site information of *GPI* can be visualized in the schematic diagram of the protein structure or the three-dimensional (3D) structure by the “Mutations” module. The data on the overall survival (OS), progression-free survival (PFS), disease-free survival (DFS), and disease-specific survival (DSS) for BRCA cases with or without *GPI* genetic alteration were obtained.

### Construction of the nomograms for BRCA survival prediction

We chose clinicopathological prognostic indexes and established a series of the table to evaluate the 1-, 3-, and 5-year OS probability of BRCA patients. In comparison with the observed actual probability *via* a calibration curve, the accuracy of the predicted probability of OS in the line chart was tested.

### Statistical analyses

Analyses were performed by using tools in Hiplot (https://hiplot.org) (18), a comprehensive and easy-to-use web service for boosting publication-ready biomedical data visualization. Gene expression data and clinical information were visualized by the R package “ggplot2” R package. The Wilcoxon signed-rank test and logistic regression were used to analyze the relationship between the clinical features of BRCA and the expression of *GPI*. Log-rank test was used to test the p-value. Spearman’s correlation analysis was used to describe the correlation between quantitative variables without normal distribution. The statistical method used was single-sample gene set enrichment analysis (ssGSEA) by R package “GSVA” of tumor-infiltrating immune cells from the gene expression profiles of BRCA samples in TCGA datasets. Ferroptosis-related genes are derived from the Liu et al. (19) systematic analysis of the aberrances and functional implications of ferroptosis in cancer. The m6A-related genes were derived from Xu’s (20) research on the molecular characterization and clinical significance of m6A modulators across 33 cancer types. The analysis methods were visualized by “ggplot2” and “pheatmap”. In all analyses, *, **, and *** indicate p < 0.05, p < 0.01, and p < 0.001, respectively.

## Results

### Gene expression analysis data

**Figure 1:**
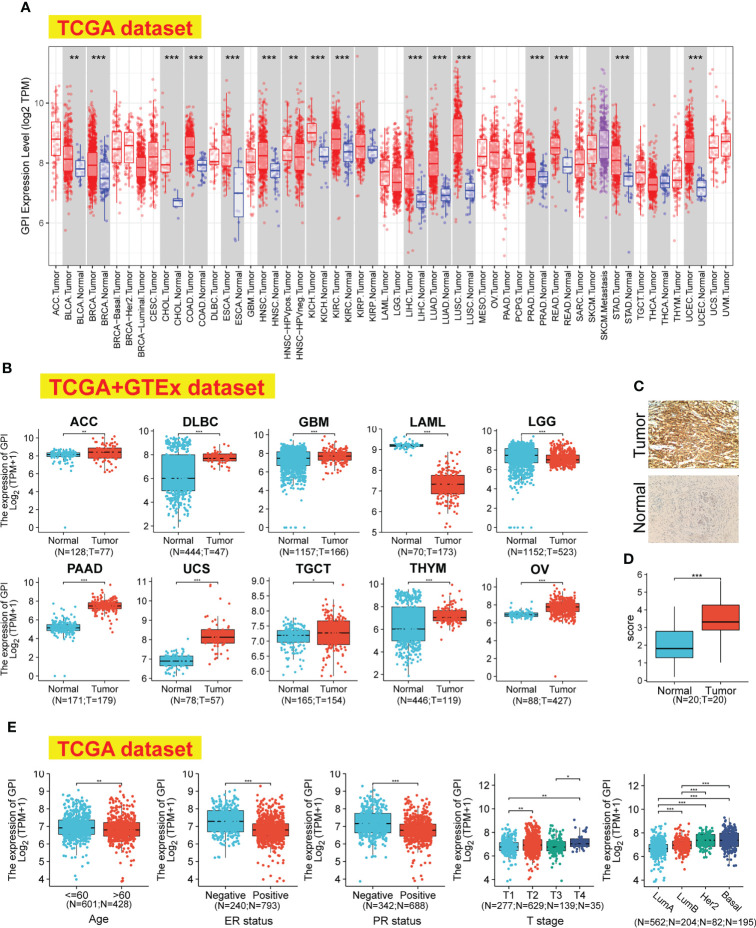
*GPI* expression levels, clinical characteristics, and immunohistochemistry in human cancer. **(A)** The expression status of the *GPI* gene in different cancers was analyzed through TIMER2. **(B)** For the type of ACC, DLBC, GBM, LAML, LGG, PAAD, UCS, TGCT, THYM, and OV, in the TCGA project, the corresponding normal tissues of the GTEx database were included as controls. **(C, D)** The expression of *GPI* in BRCA (IHC). **(E)** Expression level of *GPI* in tumor tissues from patients with different clinical characteristics in TCGA database [Age, ER status, PR status, T stage, and PAM50]. *p < 0.05; **p < 0.01; ***p < 0.001. TIMER2, Tumor Immune Estimation Resource, version 2; TCGA, The Cancer Genome Atlas; GTEx, Genotype-Tissue Expression; IHC, immunohistochemistry; ER, estrogen receptor; PR, progesterone receptor.

We applied the TIMER2 approach to analyze the expression status of *GPI* across various cancer types of TCGA. As shown in **Figure 1A**, in the tumor tissues, such as BRCA (breast invasive carcinoma), CHOL (cholangiocarcinoma), COAD (colon adenocarcinoma), ESC (esophageal carcinoma), HNSC (head and neck squamous cell carcinoma), KICH (kidney chromophobe), KIRC (kidney renal clear cell carcinoma), HCC (hepatocellular carcinoma), LUAD (lung adenocarcinoma), LUSC (lung squamous cell carcinoma), PRAD (prostate adenocarcinoma), READ (rectum adenocarcinoma), STAD (stomach adenocarcinoma), UCEC (uterine corpus endometrial carcinoma), and BLCA (bladder urothelial carcinoma) (p < 0.01), the expression level of *GPI* was significantly higher than in adjacent normal tissues (p < 0.01). Using the normal tissue of the GTEx dataset as a control, we further evaluated the expression difference of *GPI* between the normal tissues and tumor tissues (**Figure 1B**); compared with that in the corresponding normal tissues, the *GPI* expression level was significantly higher in tumor tissues such as ACC (adrenocortical carcinoma), DLBC (diffuse large B-cell lymphoma), GBM (glioblastoma multiforme), PAAD (pancreatic adenocarcinoma), UCS (uterine carcinosarcoma), THYM (thymoma), OV (ovarian serous cystadenocarcinoma), and TGCT (testicular germ cell tumors) (p < 0.05). However, the expression level of *GPI* was significantly higher in normal tissues than in tumor tissues such as LAML (acute myeloid leukemia) and LGG (brain lower-grade glioma) (p < 0.001).

We investigated the protein expression of *GPI* in BRCA and its paired adjacent tissues by immunohistochemistry (IHC). The results showed that the protein levels of *GPI* are higher in BRCA tissues than in the adjacent tissues (**Figures 1C, D**).

The association identified between *GPI* expression and clinical features in patients with BRCA is summarized in **Table 1**. As shown in **Figure 1E**, we used the Bonferroni method to correct the multiple hypothesis tests (Dunn’s test) of the significance level. Meanwhile, *GPI* mRNA expression was significantly lower in BRCA patients (age > 60) than in BRCA patients (age ≤ 60) (p.adj < 0.01). Compared with that in BRCA patients who are estrogen receptor (ER) or progesterone receptor (PR) negative, *GPI* mRNA expression was significantly lower in BRCA patients who are ER or PR positive (p.adj < 0.001). In addition, *GPI* mRNA expression was significantly lower in BRCA patients with T1 than in BRCA patients with T2/T4 (p.adj < 0.01) and was significantly lower in T3 than in T4 (p.adj < 0.01).

**Table 1:** Correlation between *GPI* expression and the clinicopathological features of the BRCA cases.

Characteristic	Low expression of *GPI*	High expression of *GPI*	p
n	541	542	
T stage, n (%)			0.004
T1	156 (14.4%)	121 (11.2%)	
T2	293 (27.1%)	336 (31.1%)	
T3	79 (7.3%)	60 (5.6%)	
T4	12 (1.1%)	23 (2.1%)	
N stage, n (%)			0.154
N0	246 (23.1%)	268 (25.2%)	
N1	185 (17.4%)	173 (16.3%)	
N2	54 (5.1%)	62 (5.8%)	
N3	46 (4.3%)	30 (2.8%)	
M stage, n (%)			0.340
M0	436 (47.3%)	466 (50.5%)	
M1	7 (0.8%)	13 (1.4%)	
Pathologic stage, n (%)			0.199
Stage I	97 (9.2%)	84 (7.9%)	
Stage II	296 (27.9%)	323 (30.5%)	
Stage III	131 (12.4%)	111 (10.5%)	
Stage IV	7 (0.7%)	11 (1%)	
Race, n (%)			0.049
Asian	22 (2.2%)	38 (3.8%)	
Black or African American	85 (8.6%)	96 (9.7%)	
White	391 (39.3%)	362 (36.4%)	
Age, n (%)			0.016
≤60	280 (25.9%)	321 (29.6%)	
>60	261 (24.1%)	221 (20.4%)	
Histological type, n (%)			<0.001
Infiltrating ductal carcinoma	340 (34.8%)	432 (44.2%)	
Infiltrating lobular carcinoma	152 (15.6%)	53 (5.4%)	
PR status, n (%)			<0.001
Negative	127 (12.3%)	215 (20.8%)	
Indeterminate	1 (0.1%)	3 (0.3%)	
Positive	387 (37.4%)	301 (29.1%)	
ER status, n (%)			<0.001
Negative	77 (7.4%)	163 (15.7%)	
Indeterminate	0 (0%)	2 (0.2%)	
Positive	438 (42.3%)	355 (34.3%)	
HER2 status, n (%)			0.482
Negative	264 (36.3%)	294 (40.4%)	
Indeterminate	7 (1%)	5 (0.7%)	
Positive	68 (9.4%)	89 (12.2%)	
PAM50, n (%)			<0.001
Normal	33 (3%)	7 (0.6%)	
LumA	354 (32.7%)	208 (19.2%)	
LumB	83 (7.7%)	121 (11.2%)	
HER2	21 (1.9%)	61 (5.6%)	
Basal	50 (4.6%)	145 (13.4%)	
Menopause status, n (%)			0.445
Pre	108 (11.1%)	121 (12.4%)	
Peri	22 (2.3%)	18 (1.9%)	
Post	362 (37.2%)	341 (35.1%)	
Anatomic neoplasm subdivisions, n (%)			0.153
Left	269 (24.8%)	294 (27.1%)	
Right	272 (25.1%)	248 (22.9%)	
Radiation therapy, n (%)			0.092
No	208 (21.1%)	226 (22.9%)	
Yes	296 (30%)	257 (26%)	
Age, median (IQR)	60 (50, 67)	56 (47, 67)	0.027

PR, progesterone receptor; ER, estrogen receptor; IQR, interquartile range.

### Kaplan–Meier survival curve analysis of the prognostic significance

**Figure 2:**
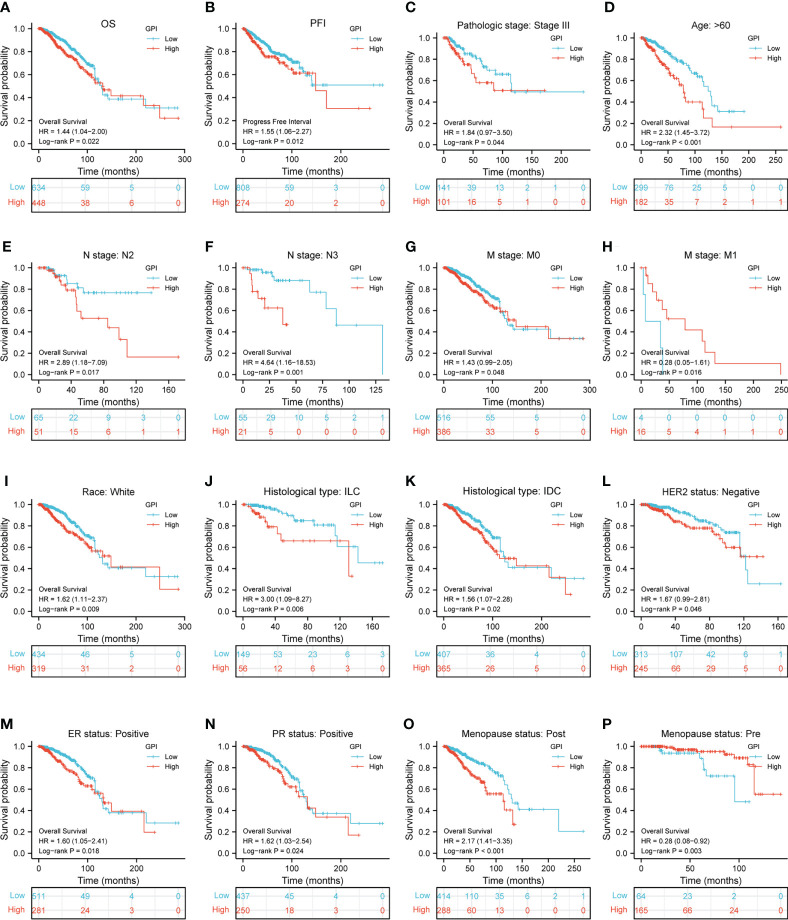
Kaplan–Meier survival curve analysis of the prognostic significance of high and low expression of *GPI* in BRCA using TCGA database. **(A, B)** Kaplan–Meier estimates of the overall survival (OS) and progression-free interval (PFI) probability of TCGA patients in all BRCA patients. Pathological staging stage III **(C)**, age greater than 60 years **(D)**, N2 **(E)**, N3 **(F)**, M0 **(G)**, M1 **(H)**, race: White **(I)**, histological type: ILC **(J)**, histological type: IDC **(K)**, HER2 status: negative **(L)**, ER status: positive **(M)**, PR status: positive **(N)**, menopause status: post **(O)**, and menopause status: pre **(P)**.

To identify whether *GPI* expression affects patient survival, we divided BRCA patients in the TCGA database into high and low *GPI* expression groups in order to perform survival analysis. The Kaplan–Meier survival analysis showed that the high expression of *GPI* was related to the poor prognosis of OS (HR = 1.44, p = 0.022) and progression-free interval (PFI) (HR = 1.55, p = 0.012) in BRCA patients (**Figures 2A, B**). In BRCA, subgroup analysis showed that a high *GPI* expression was significantly correlated with poor prognosis in the following cases: pathological stage III (HR = 1.84, p = 0.044), patients over 60 years old (HR = 2.32, p < 0.001), N2 (HR = 2.89, p = 0.017), N3 (HR = 4.68, p = 0.001), M0 (HR = 1.43, p = 0.048), race: White (HR = 1.62, p = 0.009), histological type: ILC (HR = 3.00, p = 0.006), histological type: IDC (HR = 1.56, p = 0.02), HER2 status: negative (HR = 1.67, p = 0.046), ER status: positive (HR = 1.62, p = 0.024), and menopause status: post (HR = 2.17, p < 0.001). The opposite are M1 (HR = 4.22, p = 0.005) and menopause status: pre (HR = 0.28, p < 0.003) (**Figures 2C–P**).

### Functional enrichment analysis and protein–protein interaction network

**Figure 3:**
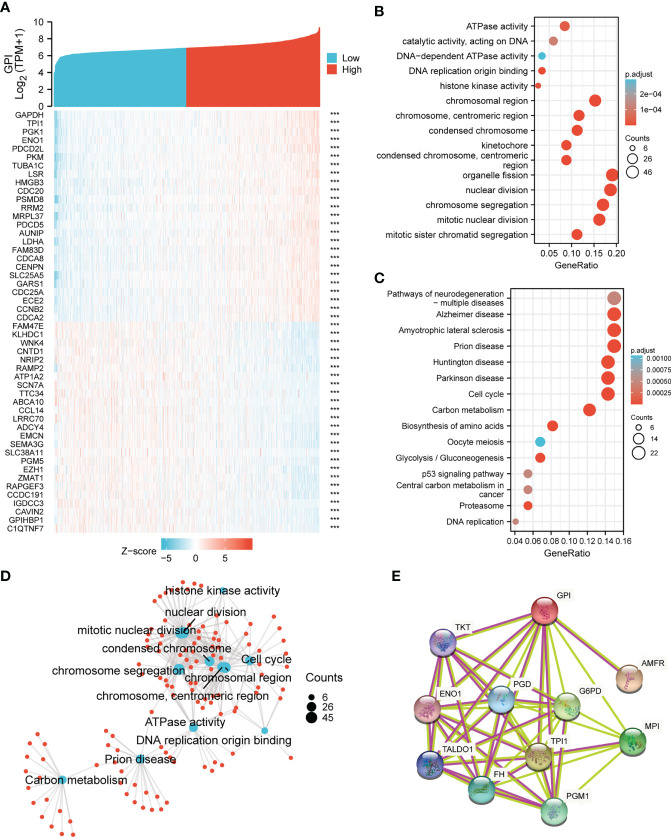
*GPI* functional and interaction network. **(A)** Heatmap showing the top 50 genes in BRCA that were positively and negatively related to *GPI*. Red represents positively related genes, and blue represents negatively related genes. **(B, C)** Gene Ontology (GO) term and Kyoto Encyclopedia of Genes and Genomes (KEGG) pathway analyses of *GPI*-related genes in BRCA. **(D)** Network of GO and KEGG enriched terms. **(E)** Protein–protein interaction (PPI) network of different expressed *GPI*. ***p < 0.001.

To understand the biological function of *GPI* in BRCA, we used the R package to detect the co-expression pattern of *GPI* in BRCA of the TCGA database. The red dot indicates the top 25 genes that were positively correlated with *GPI*, and the blue dot represents the bottom 25 genes that were negatively correlated with *GPI* (**Figure 3A**). We used the “ClusterProfiler” R package to perform functional annotation and pathway enrichment analysis of *GPI* from 600 nodes representing genes, and we found that cell cycle signaling pathways, nuclear division, and mitotic nuclear division were enriched among these genes (**Figures 3B, C**). At the same time, we made a more intuitive GO and KEGG network map to show the connection between pathways (**Figure 3D**). We performed a PPI network analysis of *GPI* at different transcription levels by STRING to study the potential interactions between them; the results showed that the PPI network diagram contained *GPI* proteins and 10 proteins that were closely related to *GPI* proteins (**Figure 3E**).

### *GPI* is closely related to cell cycle regulatory genes

**Figure 4:**
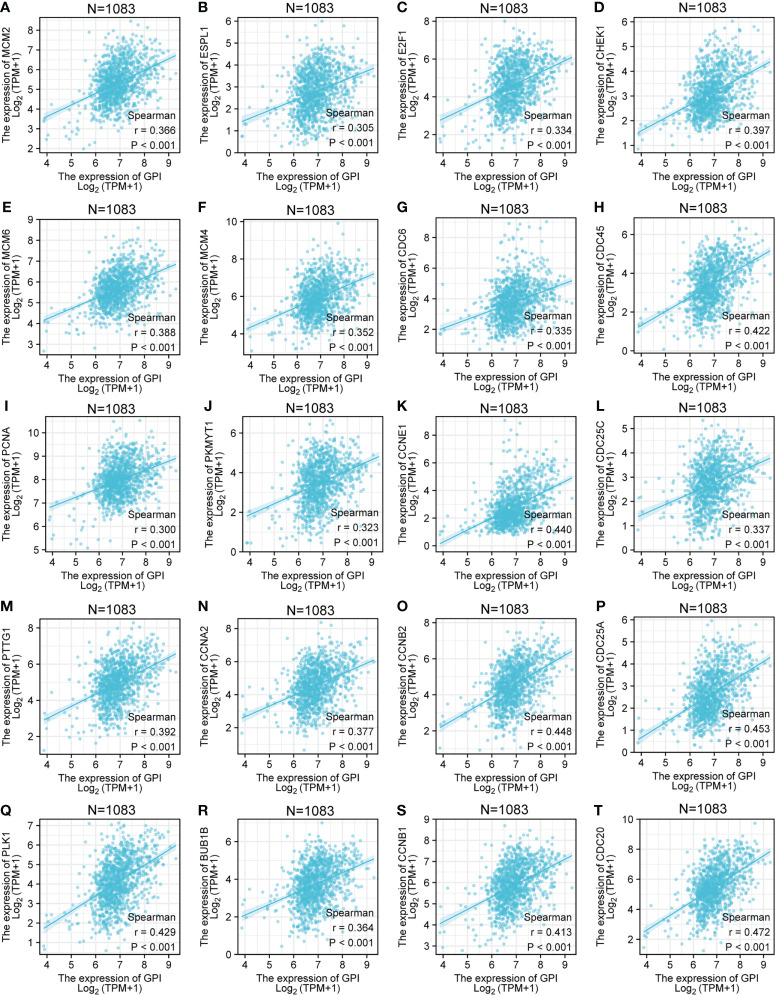
Correlation analysis between *GPI* and the cell cycle regulatory genes in BRCA. **(A)** *MCM2*, **(B)** *ESPL1*, **(C)** *E2F1*, **(D)** *CHEK1*, **(E)** *MCM6*, **(F)** *MCM4*, **(G)** *CDC6*, **(H)** *CDC45*, **(I)** *PCNA*, **(J)** *PKMYT1*, **(K)** *CCNE1*, **(L)** *CDC25C*, **(M)** *PTTG1*, **(N)** *CCNA2*, **(O)** *CCNB2*, **(P)** *CDC25A*, **(Q)** *PLK1*, **(R)** *BUB1B*, **(S)** *CCNB1*, and **(T)** *CDC20*.

The above bioinformatics analysis indicated that *GPI* was significantly enriched in the cell cycle pathway. We further analyzed the correlation between *GPI* and the cell cycle regulatory genes in TCGA-BRCA, and the results showed that the expressions of the cell cycle regulatory genes, such as *CCNA2*, *CCNB1*, *CCNB2*, *CCNE1*, *CHEK1*, *BUB1B*, *ESPL1*, *PTTG1*, *PCNA*, *PKMYT1*, *CDC45*, *PLK1*, *MCM2*, *MCM4*, *MCM6*, *E2F1*, *CDC6*, *CDC20*, *CDC25A*, and *CDC25C*, were positively correlated with *GPI* (r > 0.3, p < 0.001) (**Figures 4A–T**).

### Correlation of *GPI* expression with immune characteristics

**Figure 5:**
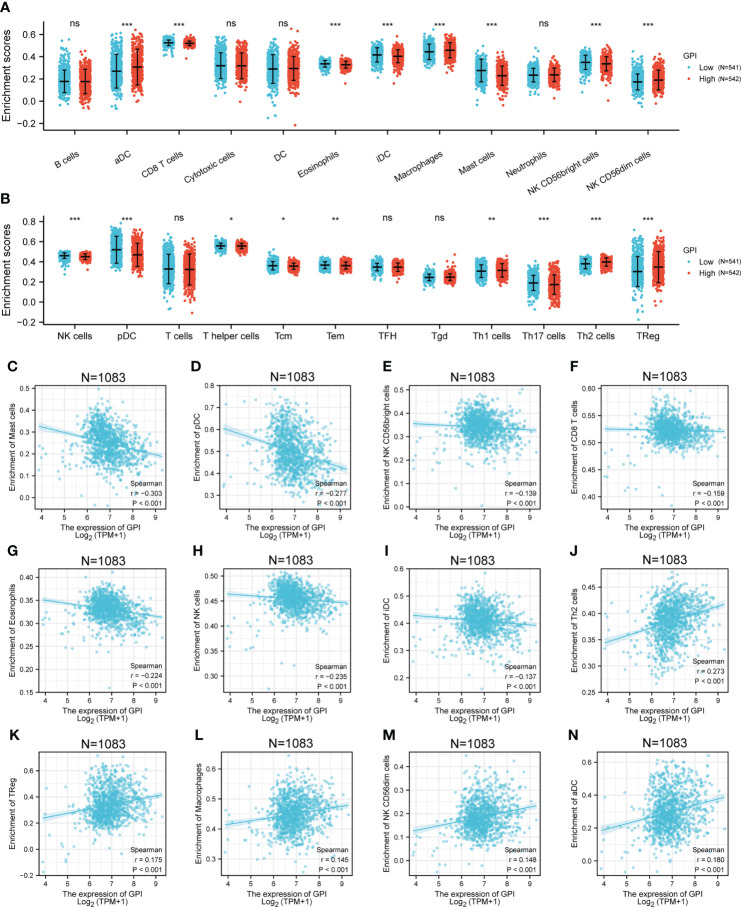
Correlation analysis of *GPI* expression and immune infiltration in BRCA. **(A, B)** Differential distribution of immune cells in patients with high *GPI* expression and low *GPI* expression. **(C–N)** Correlation between the expression level of *GPI* and immune infiltration in BRCA: **(C)** Mast cells, **(D)** pDC, **(E)** NK CD56bright cells, **(F)** CD8 T cells, **(G)** Eosinophils, **(H)** NK cells, **(I)** iDC, **(J)** Th2 cells, **(K)** TReg, **(L)** Macrophages, **(M)** CD56dim cells, and **(N)** aDC. *p < 0.05; **p < 0.01; ***p < 0.001. ns, no significance.

To explore the correlation between the expression level of *GPI* and tumor immune response, we investigated immune infiltration in BRCA with different *GPI* expression levels. The results showed that the infiltration levels of immune cells, such as CD8+ T cells, Eosinophils, iDC [immature DC], Mast cells, NK CD56bright cells, NK cells, pDC [Plasmacytoid DC], T helper cells, Tcm [T central memory], Tem [T effector memory], and Th17 cells, in BRCA patients with high *GPI* expression were significantly lower than those in patients with low *GPI* expression. In contrast, the infiltration levels of immune cells, including aDC [activated DC], Macrophages, NK CD56dim cells, Th1 cells, Th2 cells, and Treg, in BRCA patients with high *GPI* expression were significantly higher than those in patients with low *GPI* expression. In addition, the data showed that there was no significant difference in the infiltration levels of B cells, Cytotoxic cells, DC, Neutrophils, T cells, Tfh [T follicular helper], and Tgd [T gamma delta] between patients with high and low *GPI* expression (**Figures 5A–N**). Based on the results after adjusting for tumor purity, we observed that the level of *GPI* expression was significantly correlated with multiple immune markers, including *CD20*, *CD70*, *CD25*, *CD278*, *CD191*, *CD195*, *CD360*, *CD196*, *FOXP3*, *CD73*, *PD-1*, *CTLA4*, *LAG3*, *CD68*, *NOS2*, *CD163*, *CD206*, *CD86*, *CD14*, *CD57*, *KIR3DL1*, *CD7*, *CD16*, *CD1C*, and *CD141* (p < 0.05, **Table 2**).

**Table 2:** Correlation analysis between *GPI* and related genes and markers of immune cells.

Gene markers	Gene markers	rho	p	adj.p
B cell	CD19	−0.02050376	5.18E−01	9.64E−01
	CD20	0.07132238	**2.45E−02**	**2.36E−02**
	CD70	0.09297556	**3.33E−03**	**3.77E−04**
CD8+ T cell	CD8A	−0.03862236	2.24E−01	5.86E−01
	CD8B	0.01373321	6.65E−01	1.93E−01
	CD25	0.23718557	**3.44E−14**	**3.82E−19**
Tfh	CD183	−0.00218939	9.45E−01	4.20E−01
	CD185	−0.04152309	1.91E−01	5.13E−01
	CD278	0.13275243	**2.66E−05**	**9.73E−08**
Th1	CD212	0.04595712	1.47E−01	1.30E−02
	CD191	0.14618806	**3.65E−06**	**9.74E−08**
	CD195	0.03225697	3.09E−01	**4.61E−02**
Th2	CD194	−0.00678963	8.31E−01	6.10E−01
	CD365	−0.03971868	2.11E−01	2.54E−01
Th17	CD360	0.10578901	**8.31E−04**	**4.34E−06**
	IL23R	−0.01016968	7.49E−01	9.26E−01
	CD196	−0.16558630	**1.50E−07**	**2.47E−07**
Treg	FOXP3	0.14719342	**3.12E−06**	**1.29E−08**
	CD73	0.05629144	7.59E−02	**1.80E−02**
	CD127	0.02426295	4.45E−01	8.30E−02
T-cell exhaustion	PD-1	0.03466130	2.75E−01	**4.19E−02**
	CTLA4	0.11169305	**4.16E−04**	**4.69E−06**
	LAG3	0.19382176	**7.06E−10**	**8.77E−12**
Macrophage	CD68	0.11303956	**3.53E−04**	**1.22E−05**
	CD11B	−0.00015354	9.96E−01	5.99E−01
M1 macrophage	NOS2	0.08517026	**7.19E−03**	**5.41E−03**
	IRF5	0.00503338	8.74E−01	6.17E−01
M2 macrophage	CD163	0.13813891	**1.23E−05**	**2.82E−07**
	CD206	0.03600361	2.57E−01	**4.49E−02**
TAM	CCL2	0.02141342	5.00E−01	1.74E−01
	CD86	0.07329897	**2.08E−02**	**1.92E−03**
Monocyte	CD14	0.04802254	1.30E−01	**2.90E−02**
	CD33	−0.04308499	1.74E−01	4.19E−01
Natural killer cell	CD57	0.07911802	**1.25E−02**	**1.23E−03**
	KIR3DL1	0.08098825	**1.06E−02**	**1.78E−03**
	CD7	0.06127123	**5.33E−02**	**2.29E−03**
Neutrophil	CD16	0.11736297	**2.07E−04**	**2.33E−05**
	CD55	0.03867604	2.23E−01	1.81E−01
Dendritic cell	CD1C	−0.20977223	**2.34E−11**	**4.37E−12**
	CD141	−0.28466066	**5.28E−20**	**5.87E−20**

Bold values indicate p < 0.05.

Spearman’s rank correlation coefficient, rho p-value, p; q value, adj.p.

### *GPI* expression is associated with m6A RNA methylation regulators in BRCA

**Figure 6:**
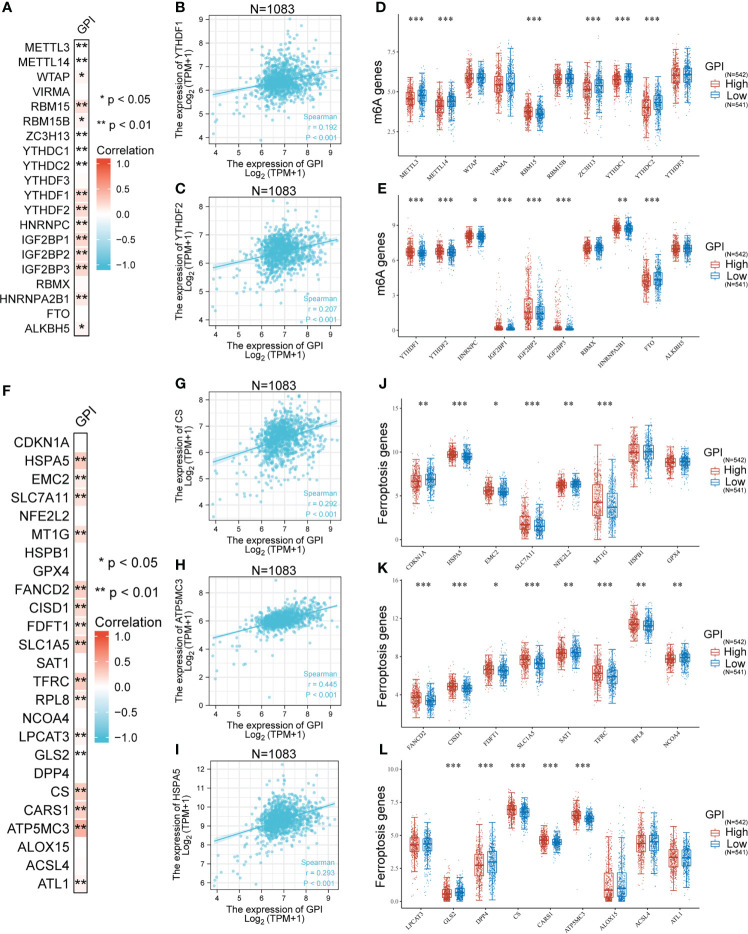
Correlation analysis of *GPI* expression, m6A, and ferroptosis-related genes in BRCA. **(A)** Heatmap showing correlation between *GPI* and m6A-related genes, **(B)** *YTHDF1*, **(C)** *YTHDF2*, and **(D, E)** differential m6A-related genes in patients with high *GPI* expression and low *GPI* expression. **(F)** Heatmap showing correlation between *GPI* and ferroptosis-related genes, **(G)** *CS*, **(H)** *ATP5MC3*, **(I)** *HSPA5*, **(J–L)** differential ferroptosis-related genes in patients with high *GPI* expression and low *GPI* expression. *p < 0.05; **p < 0.01; ***p < 0.001.

To investigate whether *GPI* expression is related to m6A modification, we analyzed the relation between the expression of *GPI* and 20 m6A-related genes in BRCA of TCGA data (**Figure 6A**). The results showed that *GPI* expression was significantly positively correlated with 11 m6A-related genes in BRCA, including *WTAP* (r = 0.073, p = 0.016), *RBM15* (r = 0.185, p < 0.001), *RBM15B* (r = 0.07, p = 0.019), *YTHDF1* (r = 0.192, p < 0.001), *YTHDF2* (r = 0.207, p < 0.001), *HNRNPC* (r = 0.118, p < 0.001), *IGF2BP1* (r = 0.227, p < 0.001), *IGF2BP2* (r = 0.173, p < 0.001), *IGF2BP3* (r = 0.232, p < 0.001), *HNRNPA2B1* (r = 0.134, p < 0.001), and *ALKBH5* (r = 0.067, p = 0.027) (**Figures 6B, C**). In addition, were divided 1,083 tumor samples into two groups, based on *GPI* expression, including 542 samples in the high-expression group and 541 samples in the low-expression group. We analyzed the expression of 20 m6A-related genes between different *GPI* expression level groups in BRCA. The results showed that, compared with that in the low *GPI* expression group, the expression of *RBN15*, *YTHDF1*, *YTHDF2*, *HNRNPC*, *IGF2BP1*, *IGF2BP2*, *IGF2BP3*, and *HNRNPA2B1* increased in the high *GPI* expression group (p < 0.05). In contrast, compared with that in the high *GPI* expression group, the expression of *METTL3*, *METTL14*, *RBM15B*, *ZC3H13*, *YTHDC1*, *YTHDC2*, and *FTO* increased in the low *GPI* expression group of *GPI* (p < 0.05) (**Figures 6D, E**). The above results indicated that *GPI* was closely related to m6A modification in BRCA.

### *GPI* expression is associated with ferroptosis genes in BRCA

A prominent role for ferroptosis in cancer development and treatment is emerging. We then explored whether there was an association between ferroptosis genes and *GPI* expression in BRCA. We analyzed the TCGA BRCA dataset to study the correlation between the expression of *GPI* and 25 ferroptosis-related genes in BRCA (**Figure 6F**). The results showed that *GPI* expression was significantly positively correlated with 15 ferroptosis-related genes in *BRCA*, including *HSPA5* (0.293, p < 0.001), *EMC2* (0.121, p < 0.001), *SLC7A11* (0.172, p < 0.001), *MT1G* (0.174, p < 0.001), *FANCD2* (0.267, p < 0.001), *CISD1* (0.233, p < 0.001), *FDFT1* (0.136, p < 0.001), *SLC1A5* (0.276, p < 0.001), *TFRC* (0.287, p < 0.001), *RPL8* (0.126, p < 0.001), *LPCAT3* (0.083, p = 0.006), *CS* (0.292, p < 0.001), *CARS1* (0.266, p < 0.001), *ATP5MC3* (0.445, p < 0.001), and *ATL1* (0.081, p = 0.007). We draw a scatter plot to show the correlation between *GPI* and ferroptosis-related genes (**Figures 6G–I**). In addition, we divided 1,083 tumor samples into two groups based on *GPI* expression, with 542 samples in the high-expression group and 541 samples in the low-expression group. We tried to analyze the differential expression of 25 ferroptosis-related genes between different *GPI* expression groups to determine whether the ferroptosis is different between high *GPI* expression level and low *GPI* expression level in BRCA (**Figures 6J–L**). The results showed that, compared with that in the low expression group, the expression of *HSPA5*, *EMC2*, *SLC7A11*, *MT1G*, *FANCD2*, *CISD1*, *FDFT1*, *SLC1A5*, *TFRC*, *RPL8*, *CS*, *CARS1* and *ATP5MC3* increased in the high expression group of *GPI* (p < 0.05). In contrast, compared with that in the high expression group, the expression of *CDKN1A*, *NFE2L2*, *SAT1*, *NCOA4*, *CLS2*, and *DPP4* increased in the low expression group of *GPI* (p < 0.05). The above results indicated that *GPI* was closely related to ferroptosis in BRCA.

### Genetic alteration of *GPI* in BRCA patients

**Figure 7:**
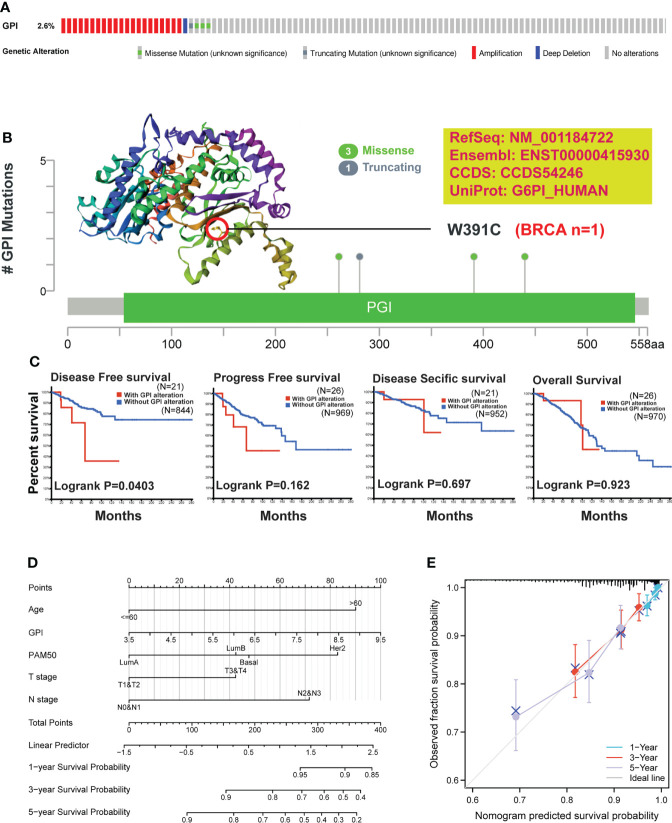
Mutation feature of *GPI* and survival prediction in BRCA. We analyzed the mutation features of *GPI* for BRCA using the cBioPortal tool. **(A)** *GPI* genome changes and **(B)** mutation site (W391C) in the 3D structure of *GPI* are displayed. **(C)** We also analyzed the potential correlation between mutation status, disease-free survival, progression-free survival, disease-specific survival, and overall BRCA. **(D)** Nomogram for predicting the 1-, 3-, and 5-year overall survival rates. **(E)** Calibration for nomogram.

To understand the mutation level of *GPI* in BRCA, we analyzed its genome and copy number. We analyzed the OncoPrint map of the *GPI* gene of BRCA patients in TCGA dataset using a cBioPortal map, and the results showed that *GPI* had 2.6% gene missense mutations, truncating mutations, amplification, and deep deletion (**Figure 7A**). In **Figure 7B**, we showed additional mutations and their location within *GPI*. We found genetic alteration and their location in the *GPI* domain. For instance, a missense mutation, W391C alteration, in the *GPI* domain, was only detected in one case of BRCA. We acquired the W391C site visualized on the 3D structure of *GPI* protein (**Figure 7B**). To see whether there is a relationship between certain genetic alterations of *GPI* and the clinical survival of patients, we systematically studied and correlated these in BRCA patients. The result showed that BRCA patients with genetic alteration of *GPI* had a poor prognosis in DFS (p = 0.0403), but not PFS (p = 0.162), OS (p = 0.923), and DSS (p = 0.697), compared with patients without *GPI* alterations (**Figure 7C**).

### Prognostic model of *GPI* in BRCA

To better predict BRCA patients’ prognosis, a nomogram was constructed based on the Cox regression analysis results using the RMS R package (**Figure 7D**). Five prognostic factor variables, *GPI* expression, age, PAM50, T stage, and N stage, were included in the model. Based on multivariate Cox analysis, a point scale was used to assign points to these variables. The straight line was drawn upward to determine the points of the variables, and the sum of the points assigned to each variable was rescaled to a range of 0–100. The points of each variable were accumulated and recorded as the total points. The probability of BRCA patient survival at 1, 3, and 5 years was determined by drawing a line from the total point axis straight down to the outcome axis. The prediction results of the nomogram calibration curve of OS were consistent with all patients’ observation results (**Figure 7E**).

## Discussion

The previous reports have indicated that *GPI* is significantly related to tumor metastasis and poor prognosis of some tumors (10–12). In this study, database analysis showed that the expression levels of *GPI* in many human tumors were frequently altered. Although the role of *GPI* in the development and prognosis of some cancers has been partially elucidated, there were few bioinformatics analyses of *GPI* expression and function in breast cancer. It was the first time to study the expression, gene alteration, regulatory pathway, and biological function of GPI in breast cancer and its influence on the prognosis in patients with breast cancer *via* bioinformatics analysis. In this study, except for LAML and LGG, the expression level of *GPI* in plenty of tumors was significantly higher than in adjacent normal tissues. IHC from our breast carcinoma specimen also demonstrated this result. In addition, our study indicated that the expression level of *GPI* was significantly related to the tumor stage, ER or PR state, and age. Similarly, Han's study (10) pointed out that the expression level of *GPI* in LUAD was significantly higher than that in paracarcinoma tissues. Rose (21) reported that serum *GPI* was often elevated in patients with breast cancer. In addition, we tried to examine the correlation of expression and prognostic of the *GPI* gene in BRCA and found that a high *GPI* expression was correlated with poor OS in BRCA. At the same time, a high *GPI* expression was correlated with poor clinicopathological characteristics, such as stage III, over 60 years old, N3, HER2 negative, and ER negative. The prediction results of the nomogram calibration curve of OS were consistent with all patients’ observation results. In agreement with our results, some studies also showed that increased expression levels of *GPI* were associated with poor prognosis in patients with clear cell-renal cell carcinoma (11), lung carcinomas (10, 22), endometrial carcinoma (23), and BRCA (24). These results strongly indicated that *GPI* may be used as a biomarker for prognosis.

Previous studies showed that *GPI* was associated with a great deal of genes or proteins (10, 25). This study aimed to clarify the underlying biological function of *GPI* and the influence of *GPI* on the prognosis of BRCA. We performed the analysis of the GO network, KEGG network, PPI network, gene alteration, and regulation of cell pathways. We found that 25 genes and 10 proteins were positively correlated with *GPI*. Further analysis suggested that the functions of these genes and proteins are mainly involved in cell cycle signaling pathways, nuclear division, and mitotic nuclear division of the cell cycle regulatory genes, including *CCNA2*, *CCNB1*, *CCNB2*, *CCNE1*, *CHEK1*, *BUB1B*, *ESPL1*, *PTTG1*, *PCNA*, *PKMYT1*, *CDC45*, *PLK1*, *MCM2*, *MCM4*, *MCM6*, *E2F1*, *CDC6*, *CDC20*, *CDC25A*, and *CDC25C*. Similarly, Han's results (10) showed that *GPI* was positively correlated with cell cycle regulatory genes in LUAD and that knockout *GPI* can prevent LUAD cells from transitioning from the G2 phase to the M phase. In addition, in this study, we observed that *GPI* was closely related to m6A RNA methylation modification in BRCA, and there was a difference in m6A RNA methylation alterations between the high *GPI* expression group and the low *GPI* expression group. Interestingly, our results showed that *GPI* in BRCA had 2.6% gene alterations, such as missense mutations, truncating mutations, amplification, and deep deletion. BRCA patients with gene alterations of *GPI* had a poor prognosis in DFS. Recent studies have verified that ferroptosis is a new form of regulated cell death and is related to the progress of BRCA (26, 27). In the present study, the database analysis revealed the correlation between *GPI* and ferroptosis-related genes in BRCA and that there was a difference in ferroptosis-related gene expression between the high *GPI* expression group and the low *GPI* expression group. The development, progress, metastasis, and clinical survival results of cancer were related to its tumor microenvironment consisting of immune cells, the extracellular matrix, and inflammatory mediators (28). Some reports indicated that *GPI*-induced arthritis was involved in a great deal of immune cells (29, 30). This study explored the correlation between the expression of *GPI* and the level of immune infiltration of BRCA. Our results showed that there was a different immune cell infiltration between the high *GPI* expression group and the low *GPI* expression group, which was similar to Han's results (10). These findings showed that *GPI* may play an important role in the tumor immune microenvironment regulation in BRCA. These data suggested that *GPI* may regulate the progression of BRCA by regulating the cell cycle, m6A RNA methylation modification, gene alteration, and tumor microenvironment.

## Conclusions

In summary, our study confirmed that the expression of *GPI* is significantly upregulated and is closely correlated to the poor prognosis of BRCA patients. *GPI* may affect the progression of BRCA by regulating the cell cycle, m6A RNA methylation modification, gene alteration, and immune infiltration, which may serve as a new prognostic biomarker for BRCA patients.

## Data availability statement

The original contributions presented in the study are included in the article/**Supplementary Material**. Further inquiries can be directed to the corresponding author.

## Ethics statement

This study was reviewed and approved by Hunan Provincial People’s Hospital, the First Affiliated Hospital of Hunan Normal University. The patients/participants provided their written informed consent to participate in this study.

## Author contributions

JNY, YZ, LZ, ST and PY and helped with data collection. JY, JZ and JY wrote the manuscript. JNY, PF, LL and CZ designed and oversaw the study. All authors contributed to the article and approved the submitted version.

## Conflict of Interest

Author ST was employed by Huazhi Medical Laboratory Co., Ltd.

The remaining authors declare that the research was conducted in the absence of any commercial or financial relationships that could be construed as a potential conflict of interest.

## Publisher’s note

All claims expressed in this article are solely those of the authors and do not necessarily represent those of their affiliated organizations, or those of the publisher, the editors and the reviewers. Any product that may be evaluated in this article, or claim that may be made by its manufacturer, is not guaranteed or endorsed by the publisher.

## References

[1] Sung H Ferlay J Siegel RL Laversanne M Soerjomataram I Jemal A. Global cancer statistics 2020: GLOBOCAN estimates of incidence and mortality worldwide for 36 cancers in 185 countries. CA Cancer J Clin (2021) 71:209–49. doi: 10.3322/caac.21660 33538338

[2] Yi J Chen S Yi P Luo J Fang M Du Y. Pyrotinib sensitizes 5-Fluorouracil-Resistant HER2(+) breast cancer cells to 5-fluorouracil. Oncol Res (2020) 28:519–31. doi: 10.3727/096504020X15960154585410 PMC7751227 32727638

[3] Yamashita T Masuda N Saji S Araki K Ito Y Takano T. Trastuzumab, pertuzumab, and eribulin mesylate versus trastuzumab, pertuzumab, and a taxane as a first-line or second-line treatment for HER2-positive, locally advanced or metastatic breast cancer: Study protocol for a randomized controlled, non-inferiority, phase III trial in Japan (JBCRG-M06/EMERALD). Trials (2020) 21:391. doi: 10.1186/s13063-020-04341-y 32381018 PMC7206765

[4] Cardoso F Castiglione M. Locally recurrent or metastatic breast cancer: ESMO clinical recommendations for diagnosis, treatment and follow-up. Ann Oncol (2009) 20 Suppl 4:15–8. doi: 10.1093/annonc/mdp115 19454439

[5] Yi J Tan S Zeng Y Zou L Zeng J Zhang C. Comprehensive analysis of prognostic and immune infiltrates for FOXPs transcription factors in human breast cancer. Sci Rep (2022) 12:8896. doi: 10.1038/s41598-022-12954-3 35614183 PMC9132954

[6] Bae YK Shim YR Choi JH Kim MJ Gabrielson E Lee SJ. Gene promoter hypermethylation in tumors and plasma of breast cancer patients. Cancer Res Treat (2005) 37:233–40. doi: 10.4143/crt.2005.37.4.233 PMC2785919 19956520

[7] Knight AL Yan X Hamamichi S Ajjuri RR Mazzulli JR Zhang MW. The glycolytic enzyme, GPI, is a functionally conserved modifier of dopaminergic neurodegeneration in parkinson’s models. Cell Metab (2014) 20:145–57. doi: 10.1016/j.cmet.2014.04.017 PMC4097176 24882066

[8] Kim JW Dang CV. Multifaceted roles of glycolytic enzymes. Trends Biochem Sci (2005) 30:142–50. doi: 10.1016/j.tibs.2005.01.005 15752986

[9] Kassahn D Kolb C Solomon S Bochtler P Illges H. Few human autoimmune sera detect GPI. Nat Immunol (2002) 3:411–2. doi: 10.1038/ni0502-411b 11976712

[10] Han J Deng X Sun R Luo M Liang M Gu B. GPI is a prognostic biomarker and correlates with immune infiltrates in lung adenocarcinoma. Front Oncol (2021) 11:752642. doi: 10.3389/fonc.2021.752642 34912709 PMC8666546

[11] Lucarelli G Rutigliano M Sanguedolce F Galleggiante V Giglio A Cagiano S. Increased expression of the autocrine motility factor is associated with poor prognosis in patients with clear cell-renal cell carcinoma. Med (Baltimore) (2015) 94:e2117. doi: 10.1097/MD.0000000000002117 PMC4652838 26579829

[12] van Veen M Matas-Rico E van de Wetering K Leyton-Puig D Kedziora KM De Lorenzi V. Negative regulation of urokinase receptor activity by a GPI-specific phospholipase c in breast cancer cells. Elife (2017) 6:e23649. doi: 10.7554/eLife.23649 28849762 PMC5576486

[13] Li T Fan J Wang B Traugh N Chen Q Liu JS. TIMER: A web server for comprehensive analysis of tumor-infiltrating immune cells. Cancer Res (2017) 77:e108–108e110. doi: 10.1158/0008-5472.CAN-17-0307 29092952 PMC6042652

[14] Tang Z Li C Kang B Gao G Li C Zhang Z. GEPIA: a web server for cancer and normal gene expression profiling and interactive analyses. Nucleic Acids Res (2017) 45:W98–98W102. doi: 10.1093/nar/gkx247 28407145 PMC5570223

[15] Szklarczyk D Gable AL Lyon D Junge A Wyder S Huerta-Cepas J. TRING v11: protein-protein association networks with increased coverage, supporting functional discovery in genome-wide experimental datasets. Nucleic Acids Res (2019) 47:D607–607D613. doi: 10.1093/nar/gky1131 30476243 PMC6323986

[16] Gao J Aksoy BA Dogrusoz U Dresdner G Gross B Sumer SO. Integrative analysis of complex cancer genomics and clinical profiles using the cBioPortal. Sci Signal (2013) 6:pl1. doi: 10.1126/scisignal.2004088 23550210 PMC4160307

[17] Cerami E Gao J Dogrusoz U Gross BE Sumer SO Aksoy BA. The cBio cancer genomics portal: an open platform for exploring multidimensional cancer genomics data. Cancer Discov (2012) 2:401–4. doi: 10.1158/2159-8290.CD-12-0095 PMC3956037 22588877

[18] Li J Miao B Wang S Dong W Xu H Si C. Hiplot: a comprehensive and easy-to-use web service for boosting publication-ready biomedical data visualization. Brief Bioinform (2022) 23:15. doi: 10.1093/bib/bbac261 35788820

[19] Liu Z Zhao Q Zuo ZX Yuan SQ Yu K Zhang Q. Systematic analysis of the aberrances and functional implications of ferroptosis in cancer. iScience (2020) 23:101302. doi: 10.1016/j.isci.2020.101302 32629423 PMC7334617

[20] Li Y Xiao J Bai J Tian Y Qu Y Chen X. Molecular characterization and clinical relevance of m(6)A regulators across 33 cancer types. Mol Cancer (2019) 18:137. doi: 10.1186/s12943-019-1066-3 31521193 PMC6744659

[21] ROSE A WEST M ZIMMERMAN HJ. Serum enzymes in disease v. isocitric dehydrogenase, malic dehydrogenase, and glycolytic enzymes in patients with carcinoma of the breast. Cancer (1961) 14:726–33. doi: 10.1002/1097-0142(199007/08)14:4&lt;726::aid-cncr2820140409&gt;3.0.co;2-1 13743254

[22] Dobashi Y Watanabe H Sato Y Hirashima S Yanagawa T Matsubara H. Differential expression and pathological significance of autocrine motility factor/glucose-6-phosphate isomerase expression in human lung carcinomas. J Pathol (2006) 210:431–40. doi: 10.1002/path.2069 17029220

[23] Wu ST Liu B Ai ZZ Hong ZC You PT Wu HZ. Esculetin inhibits cancer cell glycolysis by binding tumor PGK2, GPD2, and GPI. Front Pharmacol (2020) 11:379. doi: 10.3389/fphar.2020.00379 32292350 PMC7118906

[24] Gallardo-Pérez JC Adán-Ladrón de Guevara A Marín-Hernández A Moreno-Sánchez R Rodríguez-Enríquez S. HPI/AMF inhibition halts the development of the aggressive phenotype of breast cancer stem cells. Biochim Biophys Acta Mol Cell Res (2017) 1864:1679–90. doi: 10.1016/j.bbamcr.2017.06.015 28648642

[25] Funasaka T Hogan V Raz A. Phosphoglucose isomerase/autocrine motility factor mediates epithelial and mesenchymal phenotype conversions in breast cancer. Cancer Res (2009) 69:5349–56. doi: 10.1158/0008-5472.CAN-09-0488 PMC2875197 19531650

[26] Zhu J Dai P Liu F Li Y Qin Y Yang Q. Upconverting nanocarriers enable triggered microtubule inhibition and concurrent ferroptosis induction for selective treatment of triple-negative breast cancer. Nano Lett (2020) 20:6235–45. doi: 10.1021/acs.nanolett.0c00502 32804509

[27] Dias Lopes NM Marinello PC Sanches LJ da Silva Brito WA Lovo-Martins MI Pinge-Filho P. Patterns of cell death induced by metformin in human MCF-7 breast cancer cells. Pathol Res Pract (2020) 216:153199. doi: 10.1016/j.prp.2020.153199 32932214

[28] Wang M Chang M Li C Chen Q Hou Z Xing B. Tumor-Microenvironment-Activated reactive oxygen species amplifier for enzymatic cascade cancer Starvation/Chemodynamic/Immunotherapy. Adv Mater (2022) 34:e2106010. doi: 10.1002/adma.202106010 34699627

[29] Frey O Bruns L Morawietz L Dunussi-Joannopoulos K Kamradt T. B cell depletion reduces the number of autoreactive T helper cells and prevents glucose-6-phosphate isomerase-induced arthritis. PloS One (2011) 6:e24718. doi: 10.1371/journal.pone.0024718 21931827 PMC3169631

[30] Frey O Reichel A Bonhagen K Morawietz L Rauchhaus U Kamradt T. Regulatory T cells control the transition from acute into chronic inflammation in glucose-6-phosphate isomerase-induced arthritis. Ann Rheum Dis (2010) 69:1511–8. doi: 10.1136/ard.2009.123422 20498199

