# Supplementary material for: GPI: An indicator for immune infiltrates and prognosis of human breast cancer from a comprehensive analysis
Source: Front Endocrinol (Lausanne). 2022 Sep 28;13:995972. doi: 10.3389/fendo.2022.995972 (PMC9554491; doi:10.3389/fendo.2022.995972)
Supplement: Supplementary file 1 [file DataSheet_1.docx]

We use https://www.jianguoyun.com/ and include this link in a Word document.

https://www.jianguoyun.com/p/DVgpqf0Q2qLjChim0MwEIAA
